# Advanced Modeling and Simulation of Multilayer Spin–Transfer Torque Magnetoresistive Random Access Memory with Interface Exchange Coupling

**DOI:** 10.3390/mi15050568

**Published:** 2024-04-26

**Authors:** Mario Bendra, Roberto Lacerda de Orio, Siegfried Selberherr, Wolfgang Goes, Viktor Sverdlov

**Affiliations:** 1 Christian Doppler Laboratory for Nonvolatile Magnetoresistive Memory and Logic at the Institute for Microelectronics, TU Wien, Gußhausstraße 27-29/E360, 1040 Vienna, Austria; 2 Institute for Microelectronics, TU Wien, Gußhausstraße 27-29/E360, 1040 Vienna, Austria; orio@iue.tuwien.ac.at (R.L.d.O.); selberherr@tuwien.ac.at (S.S.); 3 Silvaco Europe Ltd., Compass Point, St Ives, Cambridge PE27 5JL, UK; wolfgang.goes@silvaco.com

**Keywords:** spintronic devices, back-hopping, spin–transfer torques, interlayer exchange coupling, micromagnetics, MRAM, synthetic antiferromagnetic

## Abstract

In advancing the study of magnetization dynamics in STT-MRAM devices, we employ the spin drift–diffusion model to address the back-hopping effect. This issue manifests as unwanted switching either in the composite free layer or in the reference layer in synthetic antiferromagnets—a challenge that becomes more pronounced with device miniaturization. Although this miniaturization aims to enhance memory density, it inadvertently compromises data integrity. Parallel to this examination, our investigation of the interface exchange coupling within multilayer structures unveils critical insights into the efficacy and dependability of spintronic devices. We particularly scrutinize how exchange coupling, mediated by non-magnetic layers, influences the magnetic interplay between adjacent ferromagnetic layers, thereby affecting their magnetic stability and domain wall movements. This investigation is crucial for understanding the switching behavior in multi-layered structures. Our integrated methodology, which uses both charge and spin currents, demonstrates a comprehensive understanding of MRAM dynamics. It emphasizes the strategic optimization of exchange coupling to improve the performance of multi-layered spintronic devices. Such enhancements are anticipated to encourage improvements in data retention and the write/read speeds of memory devices. This research, thus, marks a significant leap forward in the refinement of high-capacity, high-performance memory technologies.

## 1. Introduction

Spin–transfer torque magnetoresistive random access memory (STT-MRAM) is distinguished by its revolutionary potential in the realm of nonvolatile memory technologies, manifesting its versatility across various domains including computing-in-memory architectures [1], automotive systems [2], real-time industrial environments [3], advanced caching mechanisms [4], and dense memory configurations [5,6]. This wide-ranging applicability underscores the adaptive capacity and technological promise of STT-MRAM in meeting diverse and evolving technical demands.

At the core of an STT-MRAM lies the magnetic tunnel junction (MTJ), characterized by its intricate multilayer structure. This structure typically includes a CoFeB reference layer (RL) and a free layer (FL), separated by a MgO tunnel barrier (TB) or a non-magnetic spacer (NMS). These components are instrumental in achieving high-density memory cells through enhanced perpendicular magnetic anisotropy [7,8] and reduced cell diameters [9,10]. Such advancements are pivotal in the miniaturization of memory cells, aligning with the trend toward higher density and efficiency in memory technologies. In particular, the utilization of both interfacial and shape anisotropies within a single ferromagnetic structure, exemplified by a thick (Co)FeB layer sandwiched by MgO layers [11], provides a route to scale beyond 10nm into the single-digit nm or Xnm regime. This approach not only enhances scalability but also allows for the use of a variety of materials and stack configurations for the free layer at small dimensions while maintaining a perpendicular easy axis. This flexibility in the material and stack choice helps suppress potential detrimental effects, such as the formation of domain walls along the vertical direction, thereby contributing to the overall robustness and functionality of the device.

However, the drive toward cell size reduction and the consequent increase in current densities introduces significant reliability challenges, notably the back-hopping phenomenon, which poses a threat to memory stability [12,13]. To counteract these challenges, there has been a focused shift toward leveraging the interlayer exchange coupling (IEC) phenomenon, a critical factor in the performance and stability of memory cells, particularly in the context of complex MTJ stacks aimed at enhancing memory density [14].

In response to the demand for high-density MRAM, researchers have turned to materials with high-bulk perpendicular magnetic anisotropy to enhance stability and data retention capabilities. Among the candidate materials, L1_0_-ordered alloys like FePt and FePd, known for their high magnetocrystalline anisotropy, have shown promise in simplifying the stacking structure of recording layers compared to traditional CoFeB/MgO multilayer systems [15].

Furthermore, tetragonal phases D0_22_-Mn3Ga and D0_22_-Mn3Ge have recently received attention as potential materials for the FL in MRAM applications due to their combination of high magnetocrystalline anisotropy, low magnetic damping, and favorable thermal stability [16,17]. These attributes make D0_22_-Mn3Ga especially suitable for spin–orbit torque MRAM applications, offering pathways to enhance energy efficiency and reduce the critical current required for switching [16]. Despite these advantages, integrating D0_22_-Mn_3_Ga into existing MTJ structures poses significant challenges that need to be addressed to fully capitalize on the material’s potential in future MRAM technologies [18].

IEC, which governs the magnetic alignment between ferromagnetic layers separated by a non-magnetic spacer, has emerged as a key mechanism in spintronic devices, influencing the overall performance of STT-MRAM. The exploration and optimization of IEC are essential for advancing the stability and efficiency of MTJ-based memory technologies, providing a pathway for the development of more reliable higher-capacity memory solutions [19,20].

Furthermore, the evolution of MTJ technology, particularly within the CoFeB/MgO system, which is known for its perpendicular magnetic anisotropy, has been characterized by efforts to enhance interfacial anisotropy. This has been achieved through strategic modifications, such as adding a capping MgO layer on the CoFeB FL or integrating a MgO or NMS layer in the FL. These advancements have facilitated the scaling down of MTJs to sub-10 nm dimensions, contributing to their commercialization at nanoscale diameters by semiconductor foundries [21,22].

In light of these developments, understanding the magnetization dynamics and the role of IEC in magnetic materials becomes paramount for the precise design and optimization of STT-MRAM technologies. This knowledge is not only crucial for the accurate conceptualization of multi-layered MRAM cells, but also for enhancing the data retention and write/read speeds in memory devices, potentially enabling STT-MRAM to replace traditional memory systems like SRAM, DRAM, and flash memory in a wide area of applications, from the buffer memory to IoT/AI, automotive, and space technologies [23,24].

## 2. Micromagnetics Model

For an accurate representation of multi-layered MRAM cells, it is imperative to precisely evaluate the spin–transfer torques, which are fundamental to the memory’s functionality. Our research introduces a comprehensive modeling methodology that captures the vital physical phenomena defining the spin–transfer torques.

**Figure 1:**
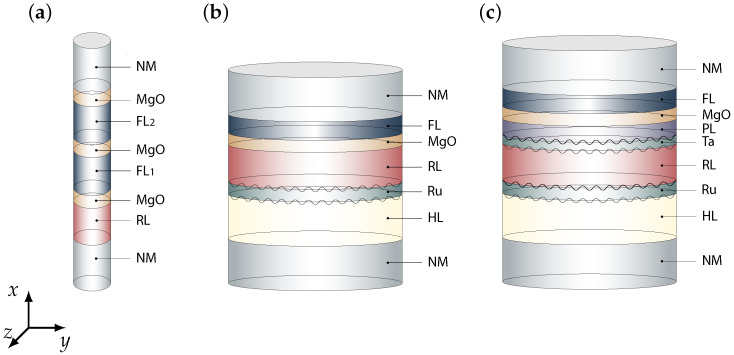
Schematic illustration to provide a clear understanding of multi-layered MRAM cell structures, each representing a distinct structural configuration, labeled as (**a**) Stack A, (**b**) Stack B, and (**c**) Stack C. To aid in distinguishing between the different components, color coding is applied. The regions where interfacial engineering is performed are denoted by black zigzag lines.

Figure 1 depicts three schematic illustrations of multi-layered MRAM cells, each with a unique structural composition, hereby referred to as Stack A, Stack B, and Stack C, respectively. In Figure 1a, representing Stack A, an ultra-scaled MRAM configuration is shown, comprising a sequential arrangement of CoFeB and MgO layers, specifically, CoFeB RL (5 nm) |MgO (0.9 nm) |CoFeB first free layer (FL_1_) (3 nm) |MgO (0.9 nm) |CoFeB second free layer (FL_2_) (3 nm) and |MgO (0.9 nm) all of which are interconnected to normal metal (NM) contacts (50 nm). The overall diameter of this configuration is 2.3 nm, highlighting the intricate layering and miniaturization achieved in this ultra-scaled design.

In Figure 1b, representing Stack B, a composite MRAM cell is depicted, featuring a layered assembly of a CoPt hard layer (HL), Ru NMS, CoFeB RL, MgO TB, and a CoFeB FL, with layer thicknesses denoted as HL (5.3 nm) |NMS_Ru_ (0.85 nm) |RL (1.1 nm) |TB (0.9 nm) and |FL (1.4 nm) respectively, linked to NM contacts (50 nm).

Figure 1c, representing Stack C, illustrates a more intricate MRAM structure, incorporating CoPt in the hard and reference layers, Ru and Ta as NMS, a CoFeB spin–polarization layer (PL), a MgO TB, and a CoFeB FL, with the sequence specified as HL (5.3 nm)|NMS_Ru_ (0.85 nm)|RL (3.2 nm)|NMS_Ta_ (0.4 nm)|PL (1.3 nm)|TB (0.9 nm)|FL (1.4 nm) again concluding in NM contacts (50 nm). The overall diameter for Stack B and Stack C is 70 nm. The simulation parameters applied across the various MRAM configurations illustrated in Figure 1 are comprehensively detailed in Table 1, with appropriate references provided in [25,26,27,28].This range aligns with the experimental values documented in NMS [29,30]. Remarkably, instances of coupling strengths over $\pm 2\;mJ/m^{2}$ have been recorded [31].

Within this framework, the Landau–Lifshitz–Gilbert (LLG) Equation (1) is numerically solved to evaluate the normalized magnetization dynamics. We utilize the finite element method (FEM) for the numerical solution. The implementation can be accessed as reported in [32]. This computational procedure is implemented in C++, taking advantage of the open-source FEM library MFEM [33].

(1) $$\frac{\partial m}{\partial t}=-\gamma m\times H_{\mathrm{eff}}+\alpha m\times \frac{\partial m}{\partial t}+\frac{1}{M_{S}}T_{S}$$

The effective magnetic field, denoted as $H_{\mathrm{eff}}$, is a crucial part of this equation, as well as a summation of the magnetic anisotropy field, the exchange field, and the demagnetization field. To compute the demagnetization field across discontinuous magnetic domains, a hybrid method combining both the boundary element method (BEM) and FEM is utilized [34]. Such a methodology offers applicability to intricate discontinuous magnetic topologies, encompassing synthetic antiferromagnets.

The respective computational implementation can be accessed as reported in [35]. Parameters are defined as follows: γ stands for the gyromagnetic ratio, $\mu_{0}$ signifies the vacuum permeability, α represents the Gilbert damping constant, M denotes the magnetization vector, which is a function of both the time and spatial position, m is the normalized magnetization vector given by $m=M/M_{S}$, and $M_{S}$ corresponds to the saturation magnetization value. The first term on the right-hand side of the LLG equation, Equation (1), expresses the precessional dynamics, wherein the magnetization vector undergoes a precessional motion around the $H_{\mathrm{eff}}$. The subsequent term characterizes a damping mechanism, which aims to orient the magnetization in congruence with the $H_{\mathrm{eff}}$. The final term is representative of the spin–transfer torque contributions.

While simulating switching dynamics in multi-layered MRAM cells, we delve into the formulation of the spin–transfer torque, denoted as $T_{S}$ and given by Equation (2). This torque arises when an electric current flows through the MTJ, undergoing polarization by its magnetic layers and becoming spin-polarized [36,37].

(2) $$T_{S}=-\frac{D_{e}}{\lambda_{J}^{2}}m\times S-\frac{D_{e}}{\lambda_{\varphi }^{2}}m\times \left(m\times S\right)$$

Here, $\lambda_{J}$ represents the exchange length, $\lambda_{\varphi }$ denotes the spin dephasing length, $D_{e}$ is the electron diffusion constant, and S symbolizes the spin accumulation.

To determine the spin accumulation, we utilize a spin and charge drift–diffusion framework, as detailed in Equation (3) through (5) [38,39,40]. This approach provides a precise description of the charge and spin transport processes within nanoscale magnetic tunnel junctions.

(3) $$D_{e}\left(\frac{S}{\lambda_{sf}^{2}}+\frac{S\times m}{\lambda_{J}^{2}}+\frac{m\times \left(S\times m\right)}{\lambda_{\varphi }^{2}}\right)=-\nabla \;\cdot \;J_{S}$$

(4) $$J_{S}=-\frac{\mu_{B}}{e}\beta_{\sigma }\left(J_{C}⊗m+\beta_{D}D_{e}\frac{e}{\mu_{B}}\left[\left(\nabla S\right)m\right]⊗m\right)-D_{e}\nabla S$$

(5) $$J_{C}=\sigma E-\beta_{D}D_{e}\frac{e}{\mu_{B}}\left[\left(\nabla S\right)m\right]$$

$J_{C}$ defines the flow of electric charge, ⊗ is the outer product, $\lambda_{sf}$ denotes the spin–flip length, σ represents the electrical conductivity, E stands for the electric field, $\beta_{D}$ and $\beta_{\sigma }$ are coefficients related to the polarization, *e* corresponds to the elementary charge of an electron, $\mu_{B}$ is the Bohr magneton, and $J_{S}$ is the spin polarization current density tensor.

Additionally, the adjustment of the charge current density is influenced by the conceptualization of the TB acts as a poor conductor, as indicated by Equation (6). The resistance of the TB varies based on the relative alignment between the adjacent layer’s magnetization [39].

(6) $$\sigma \left(\theta \right)=\frac{\sigma_{P}+\sigma_{AP}}{2}\left(1+\left(P_{RL}P_{FL}\right)\cos\theta \right)$$

$(\sigma_{P}+\sigma_{AP})/2$ is the angle-dependent portion of the conductivity, $\sigma_{AP(P)}$ is the conductivity in the anti-parallel (parallel) state, $P_{RL}$ and $P_{FL}$ are RL and FL in-plane Slonczewski polarization parameters [41], and θ is the angle between the unit magnetization vectors $m_{\mathrm{RL}(\mathrm{FL})}$. Computing the tunnel magnetoresistance (TMR) from (6) gives the Julliere expression [42], as follows:

(7) $$\mathrm{TMR}=\frac{2P_{RL}P_{FL}}{1-P_{RL}P_{FL}}$$

In the conventional FEM applied to the drift–diffusion equations, continuity is enforced for both the spin current and spin accumulation across all interfaces. To incorporate the spin current from Equation (4) into this model, we consider a low diffusion coefficient for the TB, scaled in proportion to its conductivity. We then employ this specific expression as a boundary condition at both the RL|TB and TB|FL interfaces.

(8) $$J_{C}^{\mathrm{TB}}=J_{0}\left(V\right)\left(1+P_{RL}P_{FL}\;\cdot \;\cos\theta \right)$$

(9) $$\begin{array}{c} J_{S}^{\mathrm{TB}}=-\frac{\mu_{B}}{e}\frac{J_{C}^{\mathrm{TB}}\;\cdot \;n}{1+P_{RL}P_{FL}m_{\mathrm{RL}}\;\cdot \;m_{\mathrm{FL}}}\;\cdot \;[P_{RL}m_{\mathrm{RL}}+\\ +P_{FL}m_{\mathrm{FL}}+½\left(P_{RL}^{\eta }P_{RL}-P_{FL}^{\eta }P_{FL}\right)m_{\mathrm{RL}}\times m_{\mathrm{FL}}] \end{array}$$

Equation (8) describes the relationship between the density of charge current through the TB, represented as the $J_{C}^{\mathrm{TB}}$ interface current, and the RL and FL in-plane Slonczewski polarization parameters, $P_{RL}$ and $P_{FL}$, as well as the angle θ between their magnetization vectors [43]. This equation states that the charge current density through the TB is proportional to a voltage-dependent component, $J_{0}\left(V\right)$. Additionally, the relationship is modulated by the cosine of the angle between the magnetization of the RL and the FL.

Establishing the correct boundary conditions for the density of the spin current, $J_{S}^{\mathrm{TB}}$, at the TB interfaces is of utmost importance. Equation (9) represents the boundary condition for the spin current density at the TB interfaces and is essential for accurately determining the spin current and spin accumulation in the ferromagnetic layers [39]. In this context, n refers to the normal of the interface, while $m_{\mathrm{RL}(\mathrm{FL})}$ denotes the magnetization of the RL and the FL close to the interface. The terms $P_{RL(FL)}$ are the in-plane Slonczewski polarization parameters [41]. Additionally, $P_{RL}^{\eta }$ and $P_{FL}^{\eta }$ denote the parameters linked with out-of-plane polarization [38,39].

Utilizing the boundary condition (9), we delve into the interplay of spin and charge transport and the magnetization in diverse stacks of MTJs and metallic spin valves using a comprehensive drift–diffusion methodology [38]. This method provides an in-depth analysis of the spin torques during the switching of complex multi-layered structures. An accessible computational model [44] is also available to compute spin–transfer torques in magnetic multi-layered configurations.

### Interlayer Exchange Coupling

In the exploration of layered magnetic structures, particularly those comprising multiple magnetic layers separated by NMS or TB layers, the phenomenon of IEC emerges as a pivotal factor. This magnetic interaction, pivotal in determining the magnetic orientations within such structures, is profoundly influenced by the thickness and material composition of the intervening layers. The oscillatory behavior and rapid decay of the IEC with increasing spacer thickness in metallic spacers are theoretically grounded in the Ruderman–Kittel–Kasuya–Yosida (RKKY) theory, as detailed in [45]. This theory explains the indirect interaction between localized electrons in *d*- or *f*-orbitals mediated by conduction electrons.

Conversely, in semiconducting spacers, the exchange coupling phenomena are explained by theories encompassing variable-range hopping and resonant tunneling, which are facilitated through localized electronic states within the barrier’s band gap. In the context of insulating spacers, the observed IEC, characterized by significant strengths and a lack of oscillatory behavior, is attributed to spin-dependent tunneling mechanisms [46,47].

The formulation of the free-energy density coming from IEC, as proposed by Bruno [48], is defined as follows:

(10) $$E=-J_{1}\cos\left(\Delta \phi \right)-J_{2}\cos^{2}\left(\Delta \phi \right)$$

Δϕ signifies the angle between the magnetization of adjacent magnetic layers, and $J_{1}$ and $J_{2}$ represent coefficients characterizing the nature and magnitude of the coupling. The term $J_{1}$, causing an oscillatory dependence on the spacer thickness of the RKKY interaction [49,50], determines whether the coupling is ferromagnetic (FM) or antiferromagnetic (AFM). For TBs such as MgO, a predominantly FM coupling is observed, which diminishes exponentially with the TB’s thickness, mirroring the decay in the tunneling electron wave function’s amplitude.

$J_{1}>0$ is of FM coupling, favoring a parallel (P) alignment of the magnetizations (Δϕ=0), whereas $J_{1}<0$ implies AFM coupling, favoring an anti-parallel (AP) alignment (Δϕ=π). The term $J_{2}$, while often small and, therefore, negligible, encapsulates bi-quadratic coupling terms that induce non-collinear alignment in the magnetizations [51]. Such terms are typically attributed to non-intrinsic factors, like structural defects or surface roughness [48]. When the influence of $J_{2}$ is neglected, the energy expression simplifies, solely emphasizing the bilinear coupling component:

(11) $$E=-J_{1}\cos\left(\Delta \phi \right)$$

In traditional models, IEC is often simplified as a bias field influencing the magnetic layers, an approach that facilitates a straightforward representation of its impact on the magnetization states within the layers [26,52]. However, this model’s limitation lies in its neglect of the angular variation Δϕ between the magnetizations of coupled layers, a factor that can significantly affect the IEC’s intensity during the switching processes.

To address these issues, the IEC’s boundary condition in the framework of FEM simulations can be expressed as follows, as well as enter the weak formulation of (1) on the right-hand side:

(12) $$\begin{array}{c} \frac{J_{iec}\gamma }{\mu_{0}M_{S,L}}\int_{R|spacer}m_{L}\;\cdot \;wdx+\frac{J_{iec}\gamma }{\mu_{0}M_{S,R}}\int_{spacer|L}m_{R}\;\cdot \;wdx \end{array}$$

**Figure 2:**
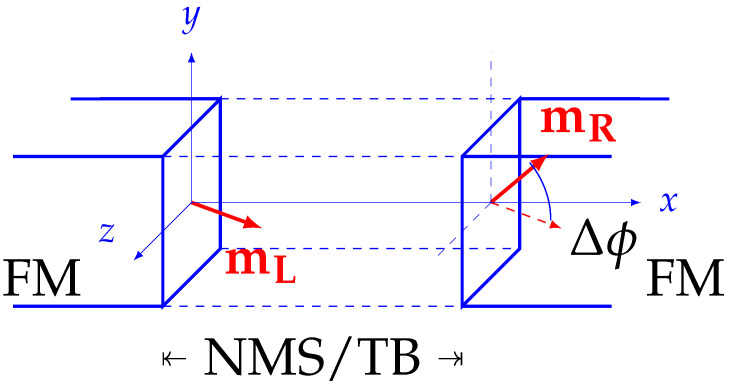
Schematic depiction of a trilayer structure composed of left and right semi-infinite ferromagnetic (FM) regions, separated by an NMS layer. The interface magnetization on the left FM interface points in an arbitrary direction, whereas the interface magnetization in the right FM forms an angle Δϕ relative to the magnetization of the left FM.

$J_{iec}$ represents the coupling’s strength, $\mu_{0}$ represents the vacuum permeability, and $M_{S,L/R}$ represent the saturation magnetizations of the left/right layers, respectively. This formulation encapsulates the coupling across the interfaces, incorporating the effects mediated through the NMS or TB layers. The interactions, represented through the normalized magnetization vectors $m_{L}$ and $m_{R}$ for the left and right layers, are visualized in Figure 2, showcasing a trilayer structure with the respective magnetizations and the interfacing angle Δϕ.

Ensuring the precision of our simulations, particularly when evaluating the boundary terms in the MFEM implementation, requires special care. This precision hinges on our knowledge of the magnetization vectors at the interfaces directly across from the calculation points. We initiate this process by establishing the coefficients for the boundary integrals, mirroring the approach used for setting up tunneling charge currents, as detailed in [40]. In scenarios involving NMS or TB coupled with IEC, our model implementation systematically evaluates each crucial location, known as the integration point. It assesses the integration points for the magnetization vectors $m_{L}$ and $m_{R}$, located on opposing sides of the spacer or barrier, selecting those that are closest to both the interface and each other.

## 3. Results and Discussion

The following sections report the results of switching simulations performed on the structures depicted in Figure 1. Due to the capability of computing the torque acting in all layers with a unified expression, the presented FE solver is suitable for the simulation of such structures.

### 3.1. Stack A

The stability of the FL can be enhanced by incorporating additional tunneling layers, making use of the perpendicular magnetic anisotropy at the interfaces with the ferromagnetic layers. Furthermore, the use of elongated layers with smaller diameters contributes to stability through shape anisotropy, as depicted in Figure 1a. The reduction of the FL diameter also improves device scalability.

**Figure 3:**
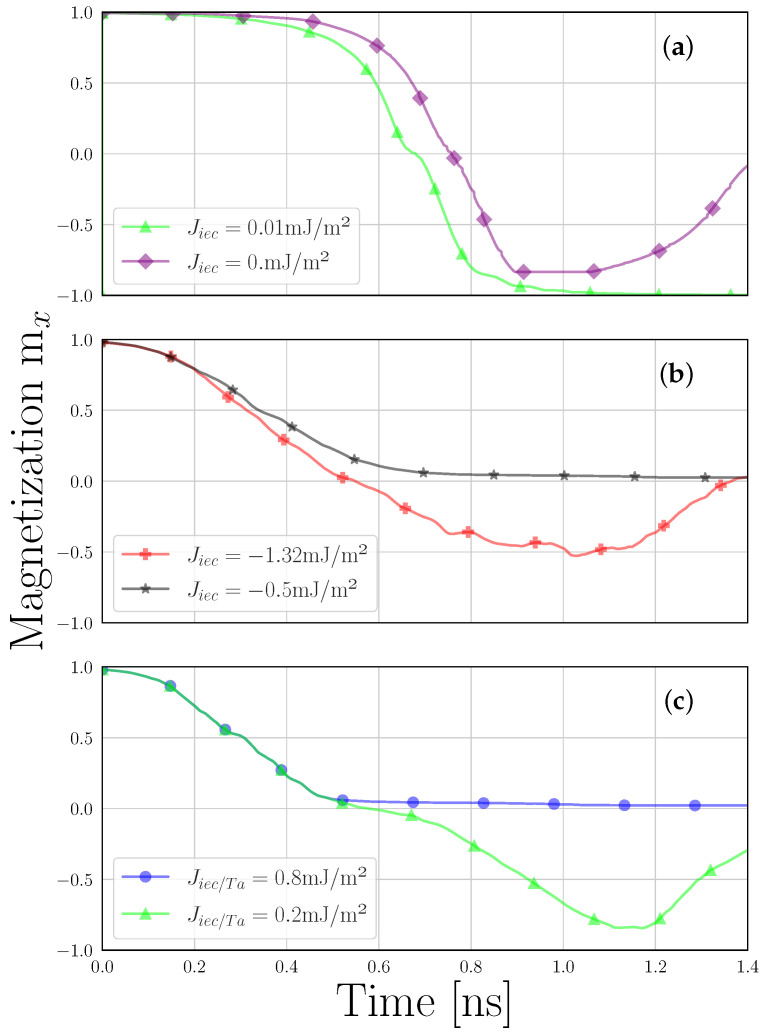
Magnetization trajectories for P to AP switching, labeled as (**a**) Stack A, (**b**) Stack B, and (**c**) Stack C.

Figure 3a presents the magnetization trajectories during the transition from P to AP alignments under a bias of 2 V. The initial magnetization set at $m_{x}=1$ represents the average magnetization direction of both FL_1_ and FL_2_, oriented positively along the *x*-axis. The magnetization reversal from P to AP is computed, highlighting the back-hopping phenomenon. Although typically undesirable in a composite FL, we demonstrated cyclic switching between four distinct states of the FL using the same current direction. This finding challenges the traditional binary perspective of the MRAM operation, offering a new multi-level functionality in ultra-scaled MRAM cells [53].

While our study confirmed that a minor FM coupling of $0.01\;mJ/m^{2}$ at the MgO TB effectively suppresses back-hopping between FL_1_ and FL_2_, and is significantly influenced by the crystalline quality, thickness, and stoichiometry of the MgO layers [54,55], it is crucial to note that our findings focused specifically on the IEC between these two layers. The potential for a similar FM coupling between the RL and FL_1_, through a MgO layer of the same thickness as that between FL_1_ and FL_2_, requires separate consideration. The presence of FM coupling between FL_1_ and FL_2_ does not automatically suggest a comparable interaction between the RL and FL_1_.

To further clarify, the mechanisms of exchange coupling through insulating layers such as MgO can be complex and depend significantly on the properties of the material. Theories such as variable-range hopping [47] and resonant tunneling through defect-generated localized electronic states in the gap of the barrier [56] have been developed to explain the exchange coupling through semiconducting spacers. This coupling through MgO notably depends on the crystalline quality of the oxide layers. Recent experimental findings, such as the sequential magnetic switching of Fe layers in interlayer exchange-coupled Fe/MgO(001) superlattices [57], continue to challenge and expand our understanding of the fundamental principles governing interlayer exchange coupling. Thus, whether similar interactions occur between the RL and FL_1_ would require specific investigation, focusing on the magnetic properties and structural details of the MgO layers involved.

### 3.2. Stack B

An alternative approach to enhancing the stability of layered structures involves incorporating synthetic antiferromagnets (SAFs) into MTJs. The depicted SAF, shown in Figure 1b, consists of a CoFeB RL, which is AFM coupled to a CoPt HL. The chosen structure, with its reduced energy barrier due to its small thickness, served as the focus for our examination of the back-hopping effect, as discussed in Hamid’s work [26].

In Figure 3b, we present the process of magnetization switching from P to AP under a bias of 2V. The initial magnetization set at $m_{x}=1$ represents the average magnetization direction of both the RL and the FL, oriented positively along the *x*-axis. At a coupling force of $-1.32\;mJ/m^{2}$, typically indicative of strong AFM coupling due to the simplified IEC [26], our observations did not align with the expected back-hopping behavior. Instead, they led to an equilibrium magnetization state of $m_{x}=0$, representing an AP configuration between the RL and the FL. This state represents the mean magnetization of the FL and the RL. Conversely, a reduced AFM coupling strength of $-0.5\;mJ/m^{2}$ results in partial back-hopping within the RL, hindered by the emergence of domain walls within both the FL and RL. This phenomenon occurs as the applied bias is insufficient at entirely inverting the magnetization.

### 3.3. Stack C

In this configuration, similar to Stack B, the inclusion of a SAF layer, complemented by a PL, aims to elevate the spin polarization. Illustrated in Figure 1c, the architecture features a CoPt RL, which is AFM-linked with a CoPt HL through a Ru NMS_**Ru**_. Additionally, a CoFeB PL is connected to the RL via FM coupling through Ta NMS_**Ta**_. The AFM bond facilitated by NMS_**Ru**_ exhibits a notable coupling force of $-1.5\;mJ/m^{2}$, indicative of a robust AFM connection. Conversely, the initial FM connection through NMS_**Ta**_, proposed at $0.8\;mJ/m^{2}$ by Devolder et al. [27], represents a substantial FM coupling. However, subsequent works by Devolder et al. [58] and Goff et al. [59] revised this FM coupling strength to $0.21\;mJ/m^{2}$, which is weaker compared to the initial FM coupling estimate.

Figure 3c depicts the transition from P to AP magnetization under a bias of 2V. The initial magnetization set at $m_{x}=1$ represents the average magnetization direction of both the PL and FL, oriented positively along the *x*-axis. With the stronger FM coupling, back-hopping is absent, suggesting that the coupling is sufficiently robust to prevent magnetization reversal in the PL. However, with the weaker FM coupling strength as later proposed, the PL’s alignment with the RL is compromised, allowing for observable magnetization reversal, with the magnetization state reaching $m_{x}=-1$.

**Figure 4:**
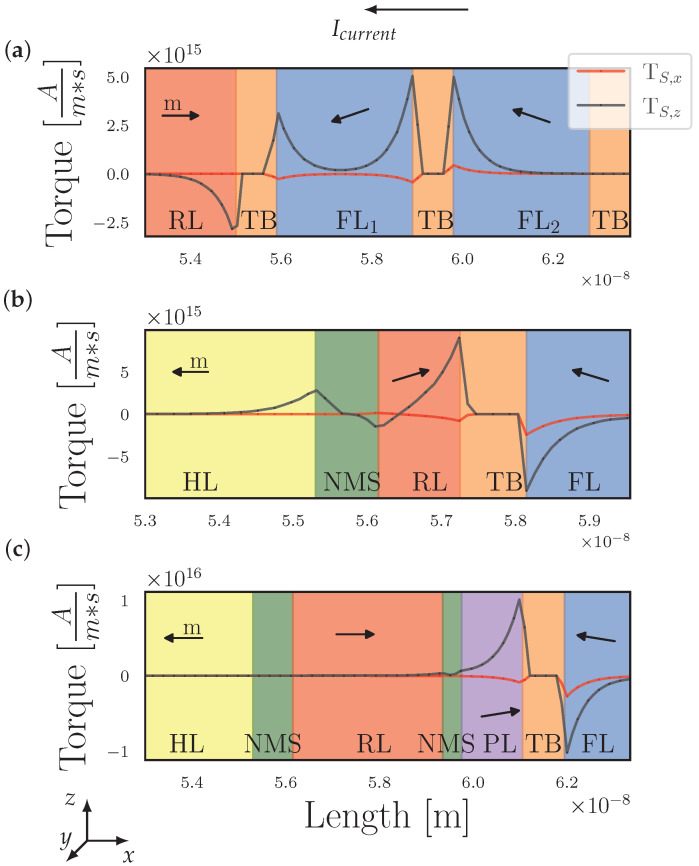
The computation of spin torque is based on the spin current boundary condition, as outlined in Equation (9). It is applied to three different configurations: Stack A (illustrated in panel (**a**)), Stack B (shown in panel (**b**)), and Stack C (depicted in panel (**c**)). These figures demonstrate the torque patterns as magnetization in FL_2_ in panel (**a**), the RL in panel (**b**), and the PL in panel (**c**) approach reversal due to back-hopping. In these diagrams, the direction of magnetization within the ferromagnetic sections is indicated by black arrows. The graphical representation reveals that $T_{S,x}$ acts as a field-like component of the spin torque along the central axis of the structure, whereas $T_{S,z}$ serves as a damping-like component. The notation $I_{current}$ is used to represent the direction of electron flow.

In Figure 4, we illustrate the spin torque calculation with Equation (4) including the spin current boundary condition Equation (9), for the scenario transitioning from P to AP as the magnetization in the FL_2_ in panel (a), the RL in panel (b), and the PL in panel (c) undergo reversal due to back-hopping.

Figure 4a shows that the torque configuration acting after the transition from P to AP is nearly obtained in Stack A. In this phase, FL_1_ and FL_2_ are slightly tilted toward the negative and positive z-axis, respectively. When the applied bias remains constant for an extended period or is increased, the magnetization in FL_2_ undergoes a magnetization reversal. Torques from RL and FL_2_ stabilize FL_1_. The torque contributions from FL_1_ initiate the magnetization reversal in FL_2_ and overcome the interface-induced uniaxial anisotropy contribution. This initiates the so-called back-hopping effect in FL_2_. As displayed in Figure 3a, even a weak FM coupling between FL_1_ and FL_2_ is sufficient to improve the switching speed, as the magnetization reversal begins more uniformly when FL_1_ and FL_2_ are coupled. Moreover, the FM coupling prevents the field-like torque from inverse FL_2_ magnetization, leading to the higher stability of the structure.

Figure 4b displays the torques after near completion of the transition from P to AP. In this setup, the torques from the HL try to keep the magnetization of the RL in the AP state. In the case of the stronger AFM coupling, the torques and the IEC are sufficient to prevent the torques from the free layer (FL) from reversing the magnetization in the RL. As seen in Figure 3b, the weaker AFM coupling is no longer strong enough to prevent the RL from reversing. As long as the bias is applied, there is a continuous interplay between the torques from the HL to keep the RL in the AP state, whereas the torques from the FL try to align it in a P orientation, leading to back-and-forth switching.

Figure 4c displays the configuration before reaching the final position in the transition from P to AP. A similar behavior as before can be observed where the FL, near complete magnetization reversal, destabilizing PL and initiating the back-hopping. Depending on the FM coupling strength between the RL and the PL, the torques are sufficient to overcome the IEC and reverse the magnetization in the PL.

## 4. Conclusions

The integration of additional tunneling layers has been shown to significantly enhance the stability of the FL by leveraging the perpendicular magnetic anisotropy at the interfaces with ferromagnetic layers. The employment of elongated layers with reduced diameters further contributes to this stability via shape anisotropy, while concurrently facilitating device scalability. The investigation of magnetization trajectories under a bias of 2V not only explains the back-hopping phenomenon but also unveils the potential for multi-level functionality in MRAM cells, challenging the conventional binary operational paradigm.

In general, stronger IEC often results in a more robust alignment of magnetic moments across the layers involved, which in turn can lead to a reduction in back-hopping. Back-hopping refers to the undesirable reversal of magnetization states in magnetic storage and sensor devices. When the IEC is strong, the magnetic moments in different layers are more tightly coupled, making it energetically unfavorable for individual layers to flip their magnetic orientation independently of the others. This increased energy barrier can suppress back-hopping by stabilizing the magnetization states that would otherwise cause these reversals. However, the correlation is not necessarily linear or straightforward. The effectiveness of IEC in suppressing back-hopping also depends on factors such as the symmetry and quality of the magnetic and non-magnetic layers, the thickness of the coupling medium, and the specific magnetic materials used. Variations in the crystalline structure or defects within the non-magnetic spacer layers can significantly affect the strength of the IEC strength, the type of coupling (FM or AFM), and, consequently, its ability to suppress or even induce back-hopping.

The subtle introduction of minor FM coupling between the FLs effectively mitigates back-hopping, as shown in our studies, with the IEC’s role being critically dependent on the MgO layer’s properties. The exploration of SAF structures within MTJs introduces a novel approach toward stability enhancement, although the expected back-hopping phenomena are not observed under strong AFM coupling, leading to an equilibrium state.

A stack configuration, featuring a SAF layer coupled with a PL, further emphasizes the importance of coupling strengths in dictating magnetization behavior, with variations in FM coupling strengths leading to differing magnetization states. The comprehensive torque analysis across various stacks underscores the intricate balance of forces at play, with the FL, the RL, and the PL experiencing varying degrees of stability and reversal tendencies based on the interplay of FM and AFM coupling strengths.

Our work not only contributes to a deeper understanding of the underlying mechanisms governing the stability and dynamics of magnetization in layered structures but also opens up avenues for the development of advanced MRAM technologies with enhanced performance and multi-level data storage capabilities.

## Figures and Tables

**Table 1:** Simulation Parameters.

LLG Parameters	Stack A	Stack B	Stack C
Saturation magnetization ($M_{S}$, HL)		$0.73\times 10^{6}\;A/m$	$0.85\times 10^{6}\;A/m$
Saturation magnetization ($M_{S}$, RL)	$0.81\times 10^{6}\;A/m$	$1.1\times 10^{6}\;A/m$	$0.8\times 10^{6}\;A/m$
Saturation magnetization ($M_{S}$, PL)			$1.1\times 10^{6}\;A/m$
Saturation magnetization ($M_{S}$, FL)	$0.81\times 10^{6}\;A/m$	$1.1\times 10^{6}\;A/m$	$1.1\times 10^{6}\;A/m$
Exchange constant ($A_{exc}$, HL)		$1.0\times 10^{-11}\;J/m$	$1.0\times 10^{-11}\;J/m$
Exchange constant ($A_{exc}$, RL)	$2.0\times 10^{-11}\;J/m$	$2.0\times 10^{-11}\;J/m$	$1.0\times 10^{-11}\;J/m$
Exchange constant ($A_{exc}$, PL)			$2.0\times 10^{-11}\;J/m$
Exchange constant ($A_{exc}$, FL)	$2.0\times 10^{-11}\;J/m$	$2.0\times 10^{-11}\;J/m$	$2.0\times 10^{-11}\;J/m$
Shape anisotropy ($K_{u}$, HL)		$7.843\times 10^{5}\;J/m^{3}$	$7.843\times 10^{5}\;J/m^{3}$
Shape anisotropy ($K_{u}$, RL)	$2.593\times 10^{5}\;J/m^{3}$	$8.501\times 10^{5}\;J/m^{3}$	$8.318\times 10^{5}\;J/m^{3}$
Shape anisotropy ($K_{u}$, PL)			$9.974\times 10^{5}\;J/m^{3}$
Shape anisotropy ($K_{u}$, FL)	$4.322\times 10^{5}\;J/m^{3}$	$9.261\times 10^{5}\;J/m^{3}$	$9.261\times 10^{5}\;J/m^{3}$
Gilbert damping constant (α, HL)		0.02	0.02
Gilbert damping constant (α, RL)	0.02	0.01	0.02
Gilbert damping constant (α, PL)			0.015
Gilbert damping constant (α, FL)	0.015	0.005	0.01
IEC ($J_{iec}$, Ru)		$-1.32\;mJ/m^{2}$	$-1.5\;mJ/m^{2}$
IEC ($J_{iec}$, Ta)			$0.8\;mJ/m^{2}$
Resistance parallel ($R_{P}$)	$4.1\times 10^{3}\;k\Omega$	1.68kΩ	1.68kΩ
Resistance parallel ($R_{AP}$)	$7.5\times 10^{3}\;k\Omega$	4.22kΩ	4.22kΩ

## Data Availability

The datasets generated during and/or analyzed during the current study are available from the corresponding author upon reasonable request.

## Abbreviations

The following abbreviations are used in this manuscript:

STT-MRAM	spin–transfer torque magnetoresistive random access memory
MTJ	magnetic tunnel junction
SRAM	static random-access memory
DRAM	dynamic random-access memory
RL	reference layer
FL	free layer
TB	tunnel barrier
NMS	non-magnetic spacer
NM	normal metal
IEC	interlayer exchange coupling
HL	hard layer
PL	spin–polarization layer
LLG	Landau–Lifshitz–Gilbert
FEM	finite element method
BEM	boundary element method
TMR	tunnel magnetoresistance
RKKY	Ruderman–Kittel–Kasuya–Yosida
FM	ferromagnetic
AFM	antiferromagnetic
SAF	synthetic antiferromagnetic
